# DGet! An open source deuteration calculator for mass spectrometry data

**DOI:** 10.1186/s13321-024-00828-x

**Published:** 2024-03-28

**Authors:** Thomas E. Lockwood, Alexander Angeloski

**Affiliations:** 1 Hyphenated Mass Spectrometry Laboratory, University of Technology Sydney, Sydney, NSW Australia; 2 National Deuteration Facility, Australian Nuclear Science Technology Organisation, Sydney, NSW Australia

**Keywords:** Deuterium, Calculator, Deuteration, Mass spectrometry

## Abstract

DGet! is an open-source analysis package written in Python for calculating the degree of deuterium enrichment in isotopically labelled molecules using mass spectrometric data. The nuclear properties of deuterium make it a valuable tracer in metabolic studies and an excellent contrast agent in nuclear spectroscopies. Determination of molecular deuteration levels is typically performed using mass spectrometry, however software options to perform these calculations are scarce. The in-house scripts and spreadsheets currently used rarely account for isotopic interferences from ^13^C or multi-isotopic elements that impact deuteration calculations. DGet! removes isotopic interferences using de-convolution and both the isotopological makeup and overall deuteration level can be accurately recovered. The software is available as command line and web applications that take a molecular formula and mass spectrometry data and output a graphical representation of the degree of deuteration as well as the distribution of partially deuterated analogues. These applications are designed to be easy to use and enable superior characterisation of deuterated molecules for users of all levels of expertise, without the limitations of techniques currently used by the majority of deuteration laboratories and researchers.

**Graphical Abstract:**

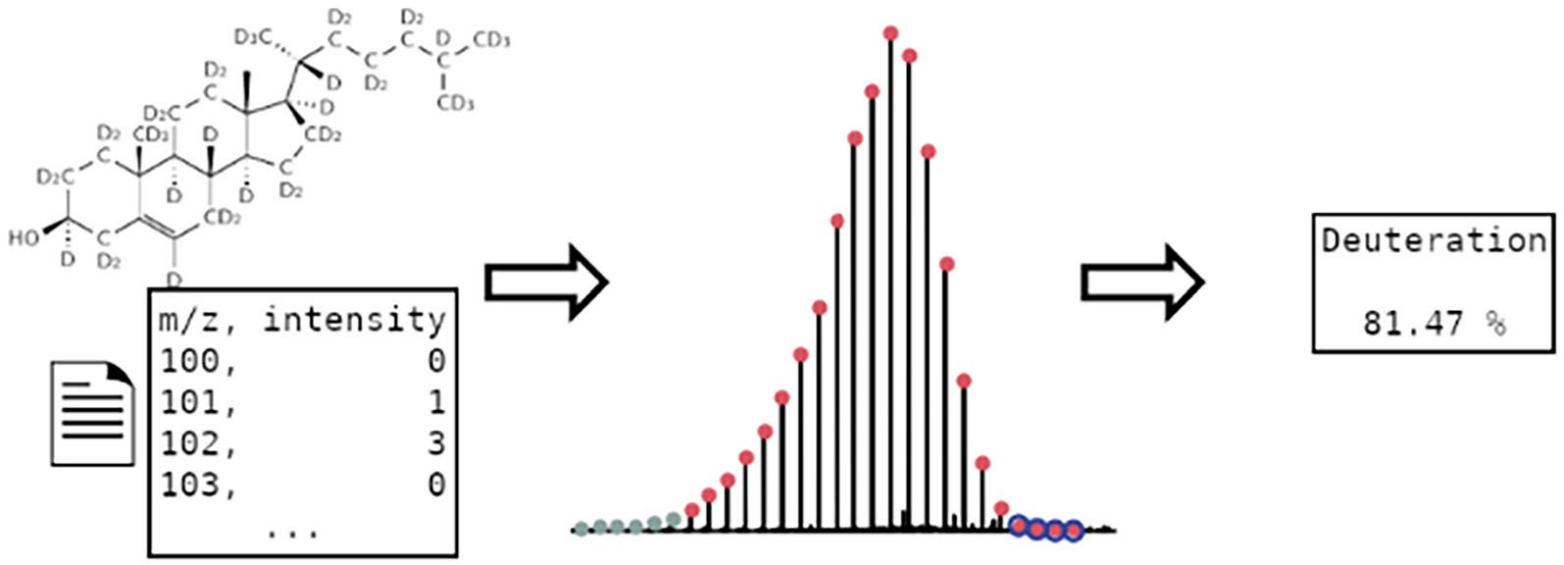

**Supplementary Information:**

The online version contains supplementary material available at 10.1186/s13321-024-00828-x.

## Introduction

**Fig. 1:**
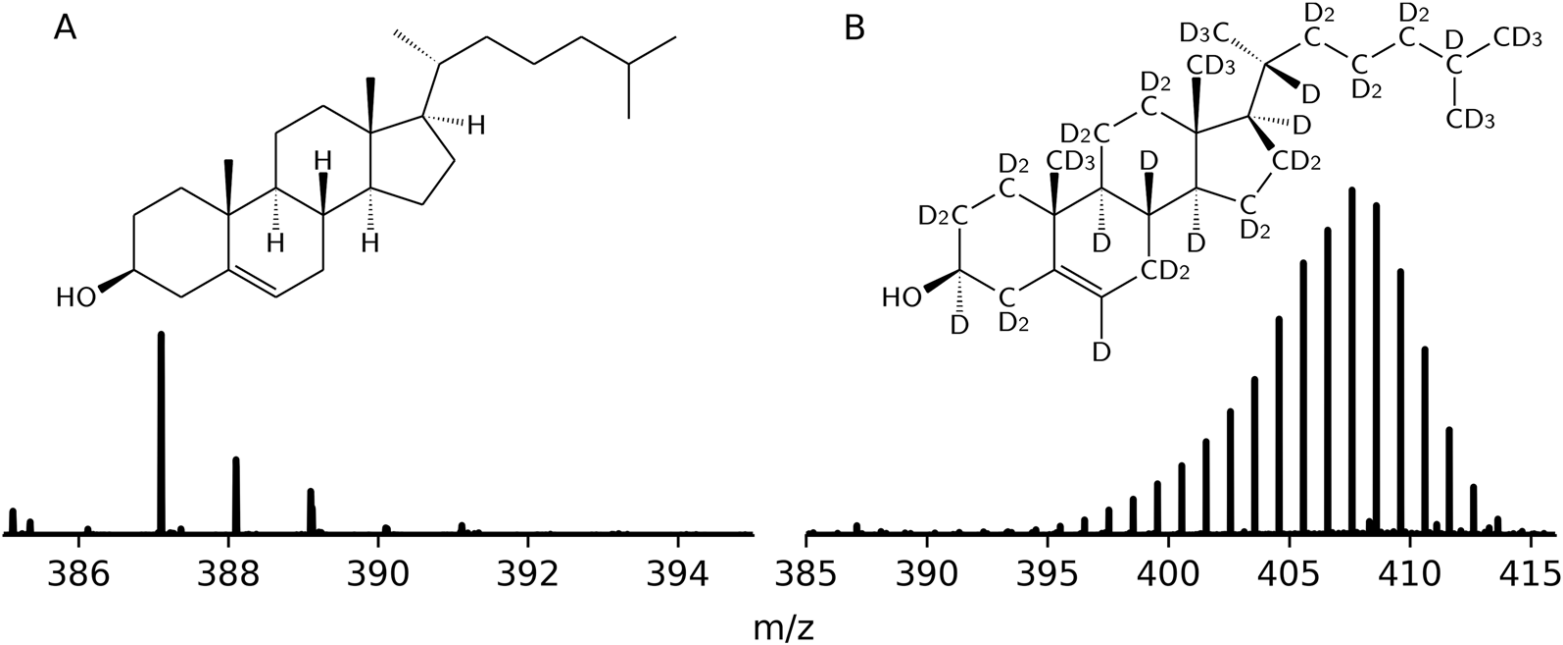
Mass spectra for undeuterated and deuterated cholesterol. Biosynthetic cholesterol obtained from Saccharomyces sp. grown in protium oxide [C_27_H_46_O] **a** and deuterium oxide [C_27_HD_45_O] **b** medium and their associated high-resolution mass spectra

Hydrogen accounts for approximately 90% of the observable universe, consisting of three naturally abundant isotopes; protium (H, ^1^H), deuterium (D, ^2^H), and tritium (T, ^3^H) [1]. Deuterium represents 0.015% of all hydrogen and has found many applications in numerous scientific areas, especially when incorporated into organic molecules [2, 3]. In these molecules, the carbon-deuterium bonds are similar to those of carbon-hydrogen but possess drastic differences in their vibrational modes and thus stability [2]. In addition to changes in vibrational modes, deuterium possesses dramatically different nuclear properties when compared to protium, such as a reduced Larmor frequency (76.773 *vs* 500.130 MHz at 11.747 T), a 40 × decrease in the incoherent neutron scattering cross section [4], and a positive coherent neutron scattering length [5]. These nuclear properties, its low natural abundance and the similarity of C-H and C-D bonds make deuterated molecules invaluable as a tracers in metabolic studies and as contrast agents in neutron and nuclear magnetic resonance spectroscopic techniques [6–15]. Deuterated molecules can be synthesized using commercially available deuterated precursors (e.g., CD_3_I, CD_3_OD, DCO_2_D, CD_3_NH_2_, LiAlD_4_, NaBD_4_) in standard chemistry techniques to produce site specific deuterated molecules [2, 16, 17]. Alternatively, deuterium can be incorporated using direct H/D exchange of protonated derivatives in D_2_O using metal catalysts [14, 18–23], or by biosynthesis using biological organisms grown in D_2_O (Fig. 1) [13, 24–26].

When performing H/D exchange reactions, the exchange of protons is typically non-specific; i.e. exchange of H to D is random and occurs universally across the molecule at each site to differing extents [27]. The variation in deuteration levels can be attributed to the steric availability of the exchangeable C-H positions, or to differing levels of stability at certain positions [27, 28]. Given the applications of these deuterated molecules, it is necessary to quantify both the overall and site-specific deuterium content to assess the degree of deuterium enrichment and to understand the distribution of isotopologues. This is especially important in cases where the deuterated analogues are used in quantitative analytical techniques or in neutron experiments as even slight hydrogen contamination will present non-negligible negative complications.

Typically, the quantification of overall deuteration is assessed using mass spectrometry (MS) by analysing the isotopic mass distribution (e.g. Figure 1b), whilst more robust techniques such as evaluation of residual ^1^H nuclear magnetic resonance signals or interpreting isotopic effects on ^13^C chemical shifts are required to determine site-specific deuteration [27, 29, 30]. While there is an abundance of software for calculating hydrogen–deuterium exchange (HDX) data [31, 32] for protein and peptide related studies, open-source tools for small molecule deuterium incorporation applications do not exist. The majority of deuteration laboratories still use inhouse scripts and spreadsheets to determine overall molecular deuteration; these existing techniques have several shortcomings such as not being able to remove the effects of interfering isotopes, produce a representation of the distribution of isotopologues, account for all possible adducts, or output a graphical representation of the deuteration fit. These shortcomings introduce difficulties in interpreting mass spectrometry data for complicated deuterated molecules containing a high number of carbon atoms, atoms with high numbers of naturally abundant isotopes, or a large distribution of partially exchanged protons.

Here we provide a simple software tool for routinely analysing and determining the total deuterium enrichment and distribution of isotopologues using mass spectrometry: *DGet!*. The DGet! software package provides users with both command line and web applications capable of analysing MS data to evaluate deuterium content.

### Implementation

The software for deuteration calculation was written in Python 3.11 using the publicly available “molmass” and “NumPy” libraries and is hosted on GitHub (https://github.com/djdt/dget) and the Python Package Index. Documentation including an API reference is also available (https://dget.readthedocs.io/en/latest). A web application has been created for users not experienced with the terminal and can be accessed through the GitHub repository or hosted locally.

**Fig. 2:**
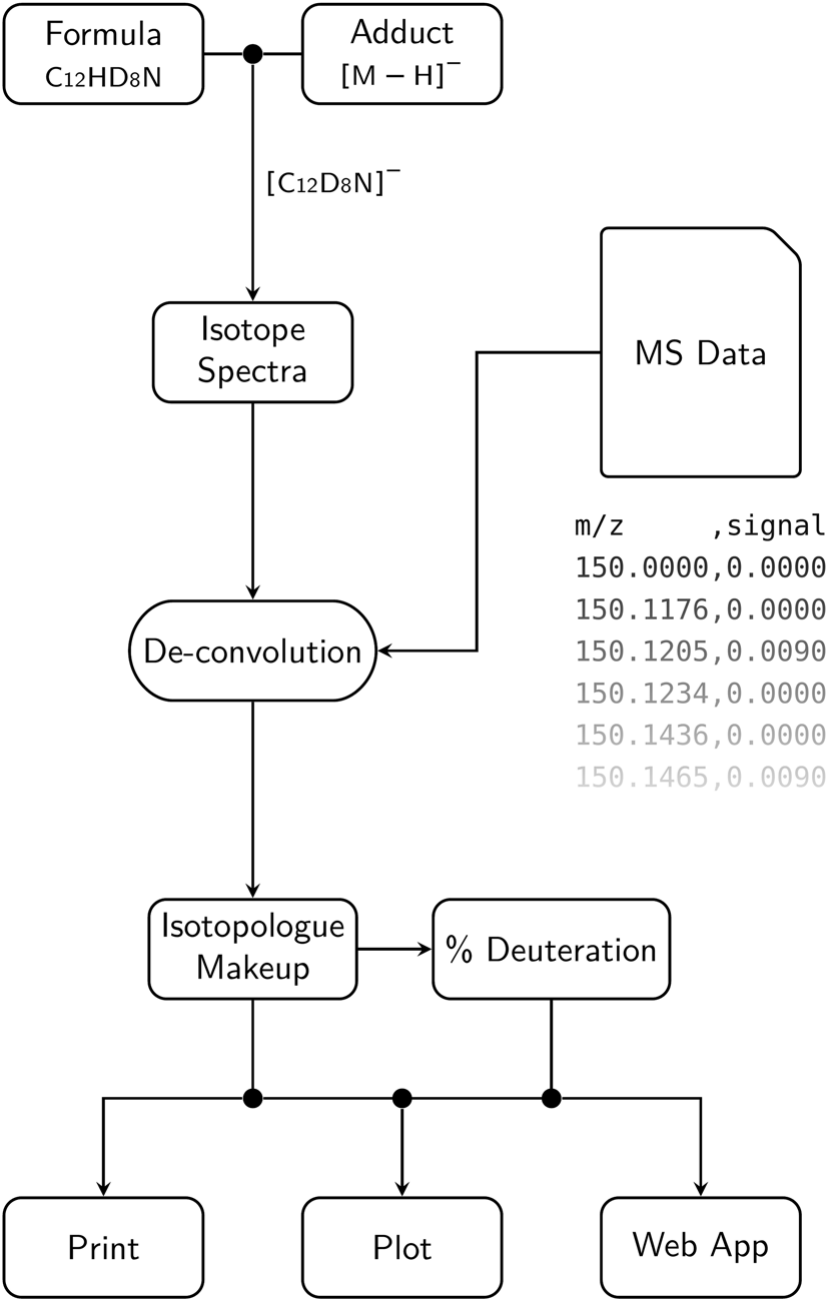
DGet! processing flow. Inputs of a formula and adduct are used to generate the isotopic spectra of the compound and target masses for all isotopologues. Peak heights or areas are extracted from the MS data at these target masses and the isotopologue makeup extracted by de-convolving, using the isotope spectra as a response function. Deuteration percentage and the makeup are then displayed to the user

The general processing workflow for DGet! is shown in Fig. 2 and requires two inputs; the molecular formula of the *fully* deuterated molecule and a file containing MS m/z and signal intensity values. MS data is supplied to the software as a delimited text file with at least two columns, one each for m/z and signal intensities. Passing these two options supports high-resolution MS (HRMS) spectra exports for the majority of vendors and options can usually be inferred from the file itself. A small module for importing Shimadzu HRMS data is included due to the different structure of the output of MS spectra.

If the adduct formed during ionisation is known then it can also be passed to the program in the form ‘[M + X]^y^’, otherwise the adduct is selected from a list of those commonly observed in electrospray ionisation [33]. In this case, the adduct with monoisotopic mass closest to that of the most intense MS peak within the expected deuteration spectrum is automatically chosen. This automatic selection can provide a good starting point for calculations but the chosen adduct should be verified by the user.

**Fig. 3:**
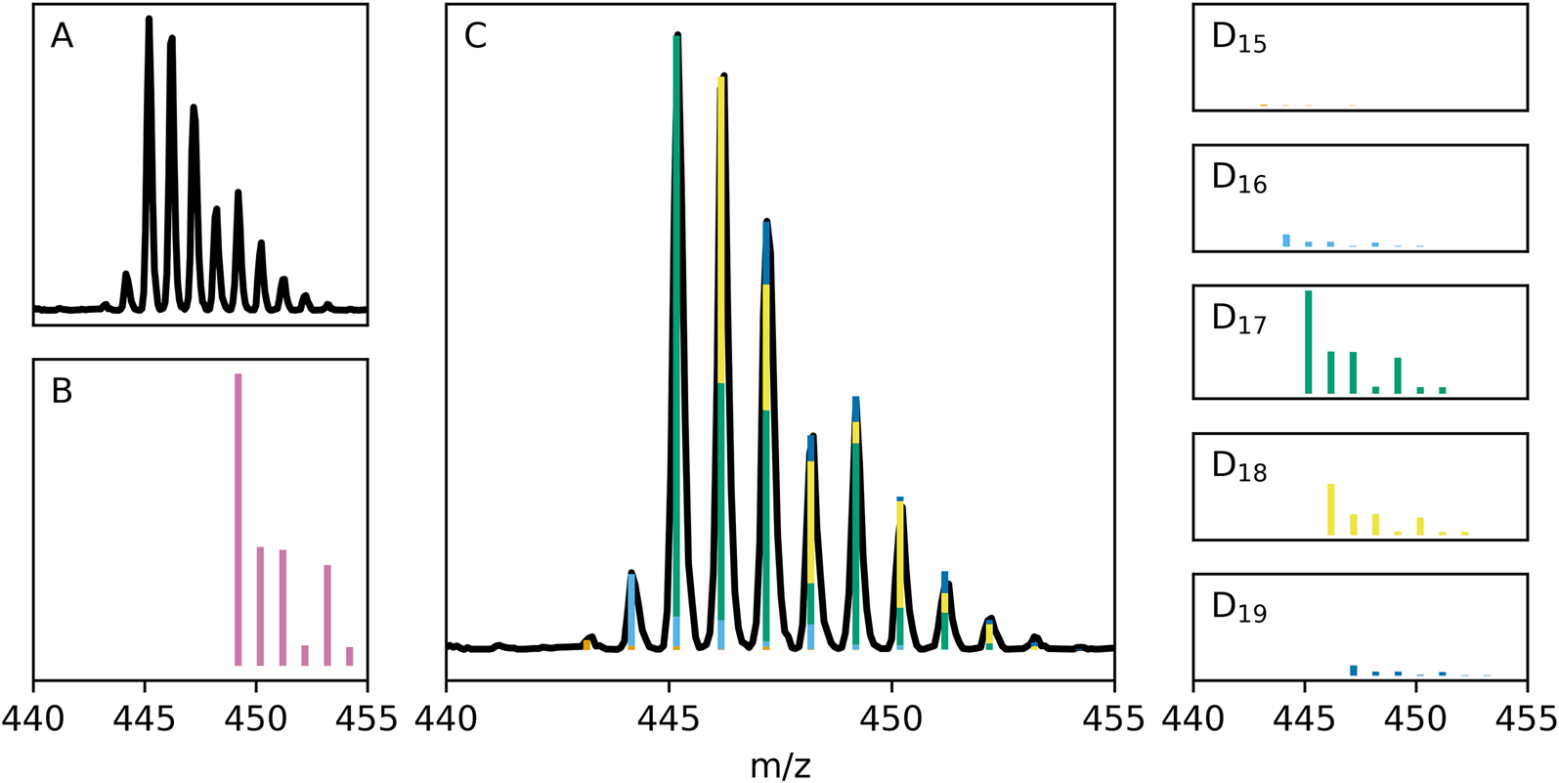
Mass and isotopologue spectra for C_20_D_28_O_8_Zr. The experimental mass spectra **A** and calculated isotopic spectra of deuterated C_20_D_28_O_8_Zr **B**, observed as the adduct [C_15_D_21_O_6_Zr + CH_3_CN]^+^. The isotopic spectrum is used as a point-spread-function to de-convolve the individual contribution of each isotopologue from the overlay mass spectra **C**. These contributions (D15-19) are then used to calculate the percentage deuteration

Calculation of the deuteration level is performed by first determining the isotopic spectra of the target MS species from the input formula and adduct. This spectrum is used as a response function ***h*** to de-convolve the individual contribution of each isotopologue from the mass spectrum. Target m/z values are created by combining the spectra of every possible isotopologue and then used to extract signals from the mass spectrum. Extraction of peak heights (or peak areas depending on the user’s preference) is performed by either selecting the maximum data point within the target region or by integration. By default, the target regions are at each target m/z value ± 0.5 Da. Probabilities for each isotopologue ${\varvec{y}}$ are then calculated by de-convolving the extracted signals ***x*** with the isotopic spectra in the frequency domain (Eq. 1). This is shown in Fig. 3, where the individual contributions of deuterium isotopolouges are extracted and displayed.

1 $${\varvec{y}} = {\mathcal{F}}^{-1}\left(\frac{\mathcal{F}({\varvec{x}})}{\mathcal{F}({\varvec{h}})}\right)$$

The percent deuteration is then calculated using Eq. 2, where *N* is the deuterium count and ${{\varvec{y}}}_{{\varvec{i}}}$ is the normalised value of ${\varvec{y}}$ for each deuteration state, $i$. The degree of deuteration is determined as the percent of the expected deuterium content taken from the number of deuterium atoms in the inputted molecular formular, not the overall H/D ratio.

2 $$\%D = \frac{100}{N}{\sum }_{i=0}^{N}i{{\varvec{y}}}_{{\varvec{i}}}$$

In molecules with many deuterium atoms the full spectra (from zero to *N* deuterium) will cover a large mass range. This exposes the calculation to interferences from contaminants or other, lower mass, adducts. To prevent these interferences, the calculation is cutoff at the lowest deuterium isotopologue where: there are two consecutive isotopologues below 1% probability and the total probability of all valid isotopologues is greater than 10%. The cutoff can also be manually assigned a deuterium count or m/z.

Issues with incorrect mass calibration of the mass spectrometer can be corrected with ease; in this case DGet! will apply a m/z offset (up to a maximum of ± 0.5 Da) to the input data to align it with the predicted spectra.

A web application for DGet! is provided in the form of a Flask module; when using the web application, a report is automatically generated which contains useful information such as overall deuteration level, distribution of partially deuterated isotopologues, and a graphical plot showing the experimental mass spectra overlayed with the predicted isotopic spectra of the target molecule (Additional file 1: Figure S9). An Illustrative guide to the usage of the web application for DGet! has been included in Additional file 1.

## Results and discussion

Compound test data was acquired from the Australian National Deuteration Facility (NDF). Each compound was provided with a molecular formula, mass spectral data and previously determined deuteration. Only compounds with clear mass spectra and an existing publication stating the percent deuteration were included. The 20 compounds are listed in Table S1 (Additional file 1) and their spectra are available in Additional file 2.

**Fig. 4:**
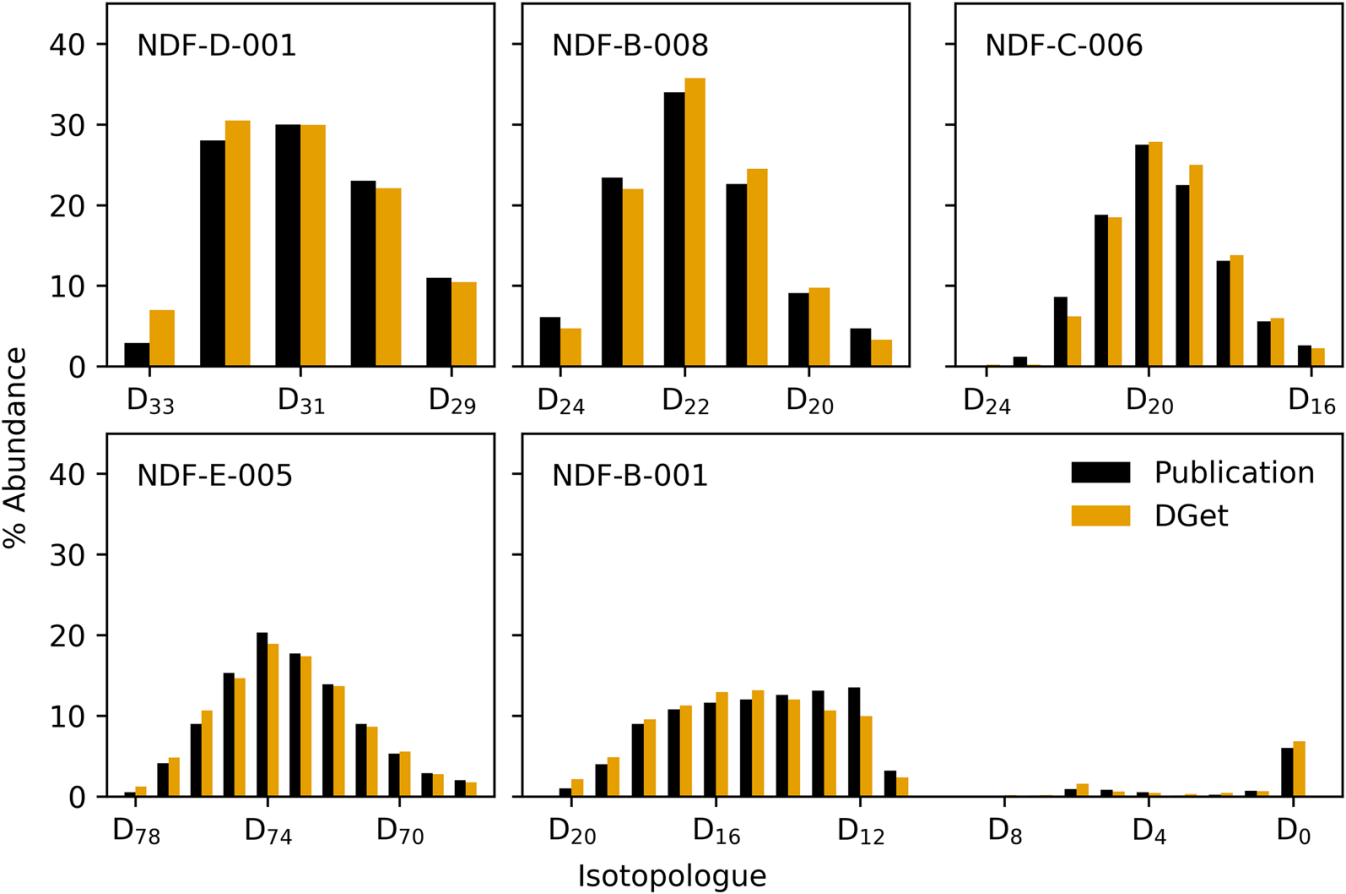
Comparison of deuterium isotopologue makeup. Mass spectra for NDF compounds with published isotopologue makeups were processed using DGet! and compared. In general, there was good agreement between the software and publications

Compounds from the NDF were analysed using DGet! to compare the calculated percentage deuteration for a representation of commonly available commercial deuterated small molecules in the literature (See additional file 1) from a wide range of classes, molecular weights and deuterium content. The average absolute difference in the reported and calculated deuteration levels for the 20 compared compounds was 0.4 ± 0.3%. These errors demonstrate the ability of DGet! to produce deuterium levels that are in excellent agreement with those previously generated using manual methods. The isotopic distribution was available for 14 compounds and compared to those retrieved using the software (Fig. 4). The average absolute error for the recovery of individual isotopologues was 2 ± 3% when using DGet! It is not clear if this represents an error or improvement over traditional methods as the methods for calculation of these states, including if isotopic interferences from ^13^C and other isotopes were accounted for, was not always available.

**Fig. 5:**
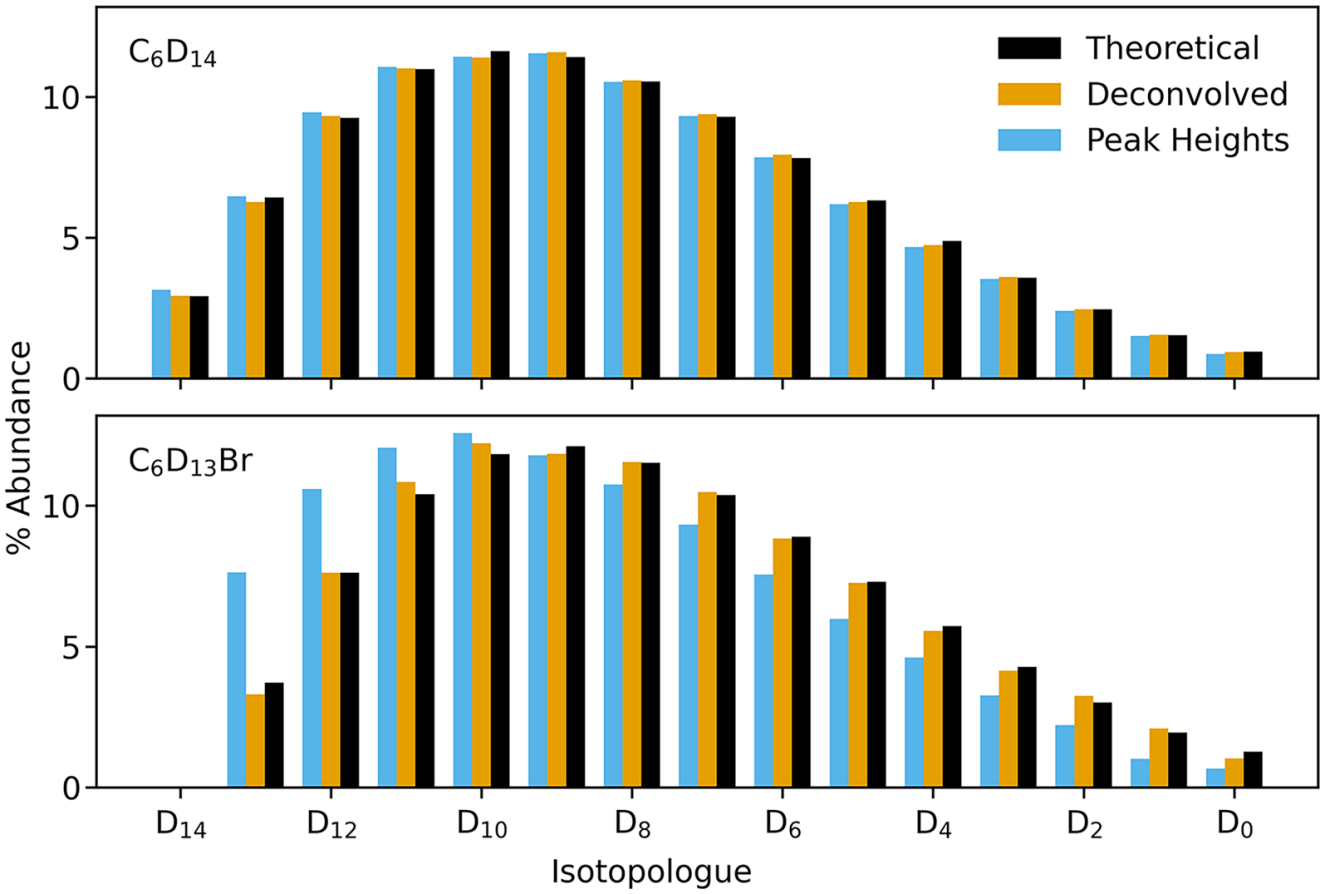
Recovery of isotopologues with and without de-convolution. The recovery of isotopologues for C_6_D_14_ is successful even without de-convolution. However, there is a large overestimation of highly deuterated isotopologues for C_6_D_13_Br when the additional Br isotopes are not compensated for

For molecules with complicated isotopic spectra, de-convolution improves the accuracy of both individual deuterium isotopologue and overall deuteration percentage. To demonstrate this hydrogen isotope exchange simulations (using Additional File 3) were performed on C_6_D_14_ and C_6_D_13_Br, with a rate ratio of 2.18 and to a final overall deuteration of 60% [34]. In Fig. 5, the isotopic distribution due to ^13^C isotopes has a small impact on the calculation of overall deuteration and both raw peak heights and de-convolution gave accurate results (60%) for C_6_D_14_ and recovery of the individual isotopologues was within 0.1%. However, once a more complicated spectra is introduced in C_6_D_13_Br, only the de-convolution is accurate. The ^79^Br and ^81^Br isotopes overlap with the deuterium spectra, shifting the apparent contributions of isotopologues and giving an apparent deuteration of 65% without de-convolution. This inaccuracy is apparent in the recovery of individual isotopologues, with a 4% error in the recovery of D_13_ when de-convolution is not used, more than double the true value.

## Conclusion

An open-source Python package was developed to address the limitations of existing deuteration calculation techniques. DGet! provides users with a simple interface in the form of command line and graphical web applications to quantify the degree of deuterium incorporation in deuterium enriched molecules using mass spectrometry data. Re-analysis of data using the new technique produced an equivalent deuterium content and distribution of isotopologues for previously reported compounds. The improved characterisation of molecules with multi-isotopic elements was demonstrated using simulations of C_6_D_13_Br and the analysis of C_20_D_28_O_8_Zr. The ease-of-use, configurability, and ability to be deployed anywhere will facilitate the adoption of the software into established deuterium enrichment calculation workflows, improving the interpretation of MS data, and advancing the field of deuteration chemistry.

### Supplementary Information

**Additional file 1: **NDF compounds list and webapp tutorial. Table of NDF compounds, molecular formulas and adducts used to validate DGet! An illustrative example of data processing using the DGet! web application.

**Additional file 2: **NDF mass spectra. Mass spectra of the NDF compounds used to validate DGet and the program parameters (formula, adduct, cutoff) used. Mass spectra of the cholesterol compounds used in Figure 1 and the webapp tutorial (Additional file 1).

**Additional file 3: **Python script used to determine theoretical makeup.

## Data Availability

The dataset supporting the conclusions of the article are included within the article and its additional files.
