# Supplementary material for: DGet! An open source deuteration calculator for mass spectrometry data
Source: J Cheminform. 2024 Mar 28;16:36. doi: 10.1186/s13321-024-00828-x (PMC10976818; doi:10.1186/s13321-024-00828-x)
Supplement: Supplementary file 1 — Additional file 1: NDF compounds list and webapp tutorial. Table of NDF compounds, molecular formulas and adducts used to validate DGet! An illustrative example of data processing using the DGet! web application. [file 13321_2024_828_MOESM1_ESM.docx]

**DGet! An open source deuteration calculator for mass spectrometry data.**

Thomas E. Lockwood ^a^, Alexander Angeloski ^b*^

Table S1. **Compounds used to test DGet!** Catalogue numbers refer to the National Deuteration Facility Product List, available at https://www.ansto.gov.au/media/5698/download.

| Catalogue | Formula (Ion) | Adduct | Deuteration | DOI |
| --- | --- | --- | --- | --- |
| NDF-A-009 | C_8_D_15_HO_2_ | [M-H]^-^ | 96.3 | 10.1016/j.jcis.2014.11.026 |
| NDF-A-011 | C_12_D_23_HO_2_ | [M-H]^-^ | 97.3 | 10.1016/j.jcis.2014.11.026 |
| NDF-A-012 | C_14_HD_27_O_2_ | [M-H]^-^ | 97.7 | 10.1016/j.jcis.2014.11.026 |
| NDF-B-001 | C_26_D_20_N_2_ | [M+H]^+^ | 68.0 | 10.1016/j.tetlet.2011.12.032 |
| NDF-B-002 | C_54_H_12_D_24_N_4_ | [M]^+^ | 94.5 | 10.1016/j.tetlet.2011.12.032 |
| NDF-B-004 | C_38_D_32_N_2_ | [M+H]^+^ | 91.0 | 10.1002/admi.201700872 |
| NDF-B-008 | C_44_H_8_D_24_N_2_ | [M]^+^ | 90.7 | 10.1002/admi.201600184 |
| NDF-B-030 | C_12_HD_8_N | [M-H]^-^ | 94.4 | 10.1016/j.tetlet.2011.12.032 |
| NDF-B-034 | C_36_D_24_N_2_ | [M+H]^+^ | 95.5 | 10.1021/acsami.1c5940 |
| NDF-B-040 | C_6_D_6_ClN | [M+H]^+^ | 95.0 | 10.1088/1361-648X/aabdf9 |
| NDF-B-045 | C_8_HD_15_O_2_ | [M-H]^-^ | 96.9 | 10.1039/c9tc05322k |
| NDF-B-052 | C_8_HD_7_O_3_ | [M-H]^-^ | 93.3 | 10.1002/chem.202201366 |
| NDF-C-006 | C_14_H_4_D_24_O_6_ | [M+Na]^+^ | 81.8 | 10.1111/febs.14345 |
| NDF-D-001 | C_25_H_15_D_33_N_2_O_3_ | [M+Na]^+^ | 93.5 | 10.1016/j.jcis.2018.09.046 |
| NDF-E-003 | C_44_H_18_D_66_NO_8_P | [M+H]^+^ | 93.3 | 10.1016/j.colsurfb.2019.01.040 |
| NDF-E-005 | C_48_H_18_D_78_NO_8_P | [M+Na]^+^ | 94.0 | 10.1016/j.chemphyslip.2014.04.004 |
| NDF-E-007 | C_40_H_71_D_9_NO_8_P | [M+H]^+^ | 99.0 | 10.1016/j.colsurfb.2019.01.040 |
| NDF-H-002 | C_21_H_2_D_38_O_4_ | [M+Na^]+^ | 94.1 | 10.1021/acs.langmuir.9b00647 |
| NDF-H-006 | C_51_H_5_D_93_O_6_ | [M+Na^]+^ | 97.9 | 10.1016/j.chemphyslip.2019.02.011 |
| NDF-J-007 | C_22_H_2_D_43_NO_2_ | [M+Na]^+^ | 98.5 | 10.1021/acs.jpclett.6b01173 |

**Illustrative example for analysing deuterium content using the DGet! web-app.**

The basic protocol for analysing mass spectroscopic data using the web app (Figure S1) for DGet! (available at <https://github.com/djdt/dget>) is presented below. Multiple demo data sets with known deuterium content and distributions are available for testing the software in the “tests/data” folder of the GitHub repository and in the ESI. Here we will determine the overall deuteration conversion and distribution of isotopologues for cholesterol-*d*45 (C_27_HD_45_O as [M+H-H_2_O]^+^) using the spectra presented in Figure 1 of the manuscript main text.


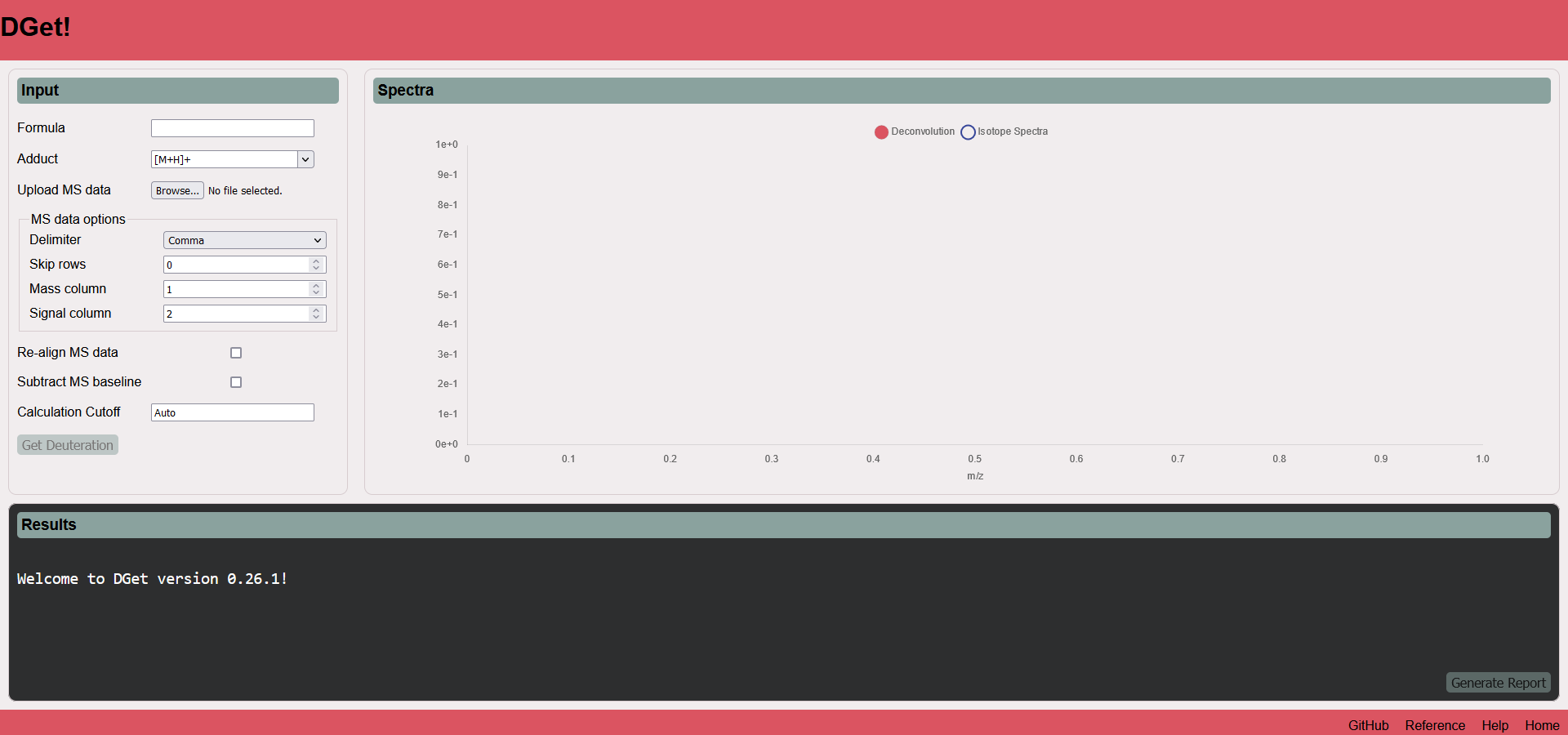


Figure S1: Web application for DGet! home page

The user begins by loading in their mass spectroscopic data in the form of a .txt file containing m/z and intensity columns and an appropriate molecular formula for their molecule of interest (Figure S2).


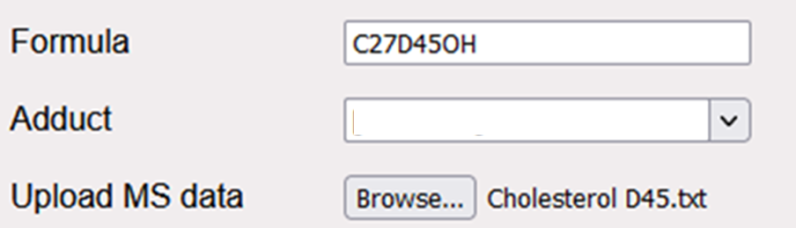


Figure S2: Location for input of the experimental data, adduct information, and molecular formula.

DGet! will automatically determine the file format, or the user can amend the information as appropriate (Figure S3):


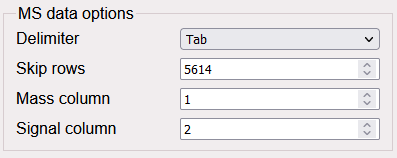

Figure S3: MS data options for the input MS data

Once the data and formula have been input, the user will need to set the desired adduct (Figure S4). Options for the most frequently observed adducts are included in the “Adduct” drop-down menu, however any adduct can be input by manually entering it in the “Adduct” menu box. Alternatively, the user can select “Auto” which will choose the adduct with monoisotopic mass closest to that of the most intense MS peak within the expected deuteration spectrum. Note that we advise the user to visually verify the fit and output and interpret the adduct selection with caution (e.g., in cases where the adduct charge cannot be physically observed in the MS measurement polarity).


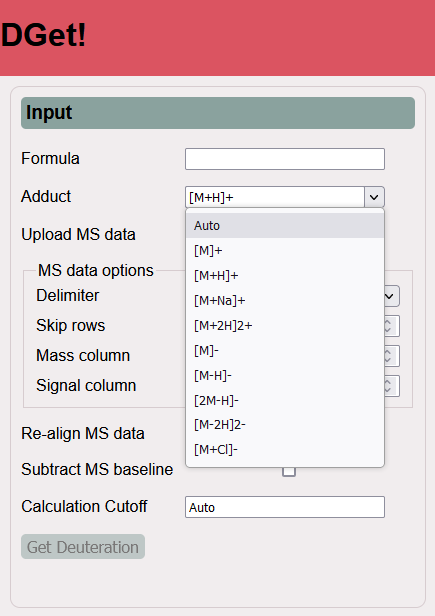


Figure S4: Default adducts available in DGet!

The user then has the choice to select or modify the program options (Figure S5):

- Re-align MS data will correct for issues with incorrect mass calibration of the mass spectrometer by applying up to a ± 0.5 Da m/z offset to the input data to align it with the predicted spectra.
- Subtract MS baseline will subtract the baseline MS intensity; this option does not change the calculated %D or distribution of isotopologues.
- Calculation Cutoff will change the cutoff for calculating %D or distribution of isotopologues (Figure S6). By default, “Auto” will cutoff at the lowest deuterium isotopologue where there are two consecutive isotopologues below 1% probability and the total probability of all valid isotopologues is greater than 10%.


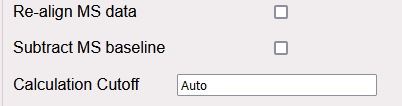


Figure S5: Calculation options available in DGet!


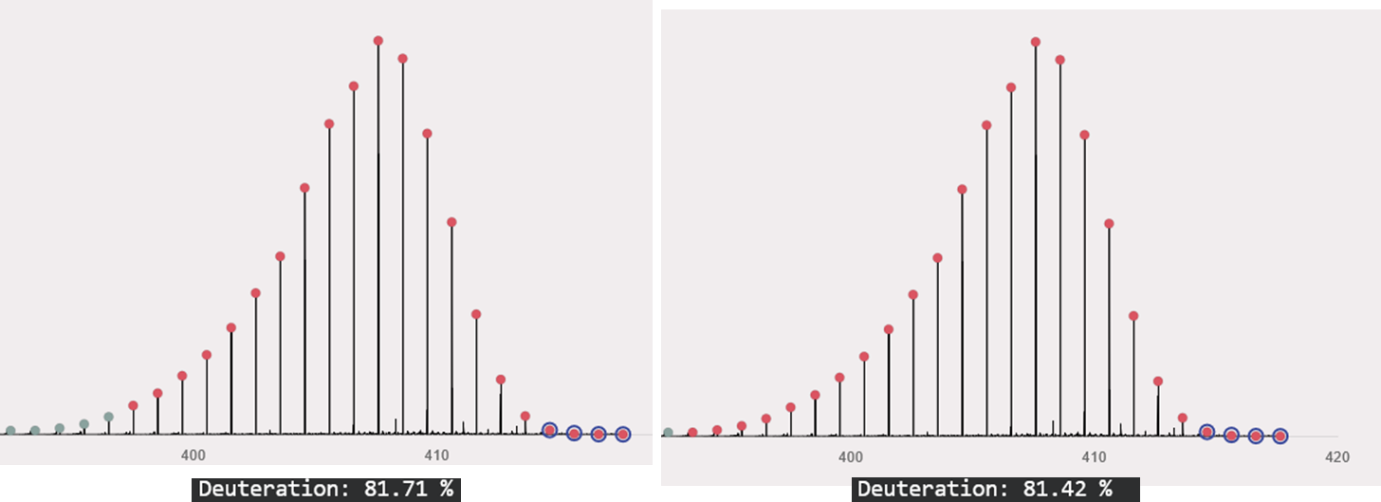


Figure S6: The use of automatic cutoff (left) and user supplied cutoff (right) showing effect of cutoff on the calculated % deuteration.

Once all these steps have been followed, the user simply presses “Get Deuteration” and DGet! will provide a graphical summary of the calculated values and predicted *vs* observed spectra (Figure S7):


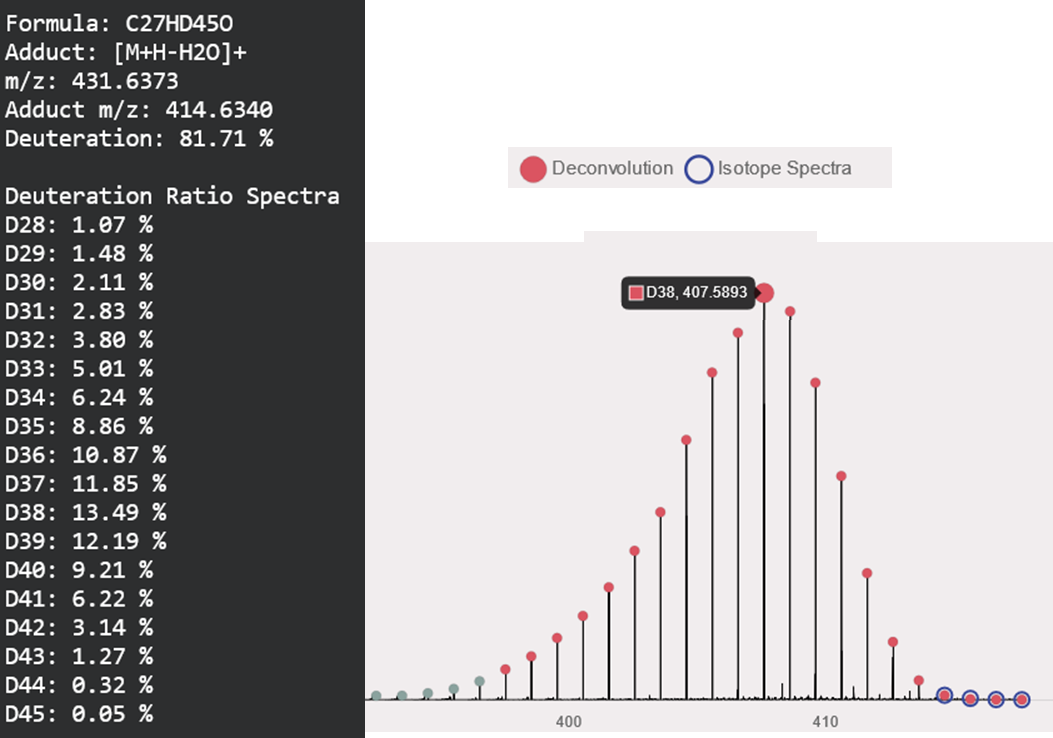


Figure S7: Graphical representation of output provided by DGet! after performing a “Get Deuteration” calculation. Figure has been modified for clarity.

Users can obtain information about the fit of the spectra by interpretation of the coloured circles; solid-coloured red circles represent peaks used for the deconvolution; solid green-grey circles represent data that is within the range of possible deuteration states based on the input formula. By hovering the mouse pointer over the circles, the user is given a description of the peak. The isotopic spectra used for deconvolution, from the contribution of non-deuterium isotopes (i.e., ^13^C), is displayed as blue outlined circles and is scaled to the 100% D peak.

The entire analysis can be saved in a .pdf form by pressing “Generate Report” in the bottom right-hand side (Figure S8) and then printing the generated report (Figure S9). A custom name or compound ID and general notes can be added in the “Name / ID” and “Notes” text fields.

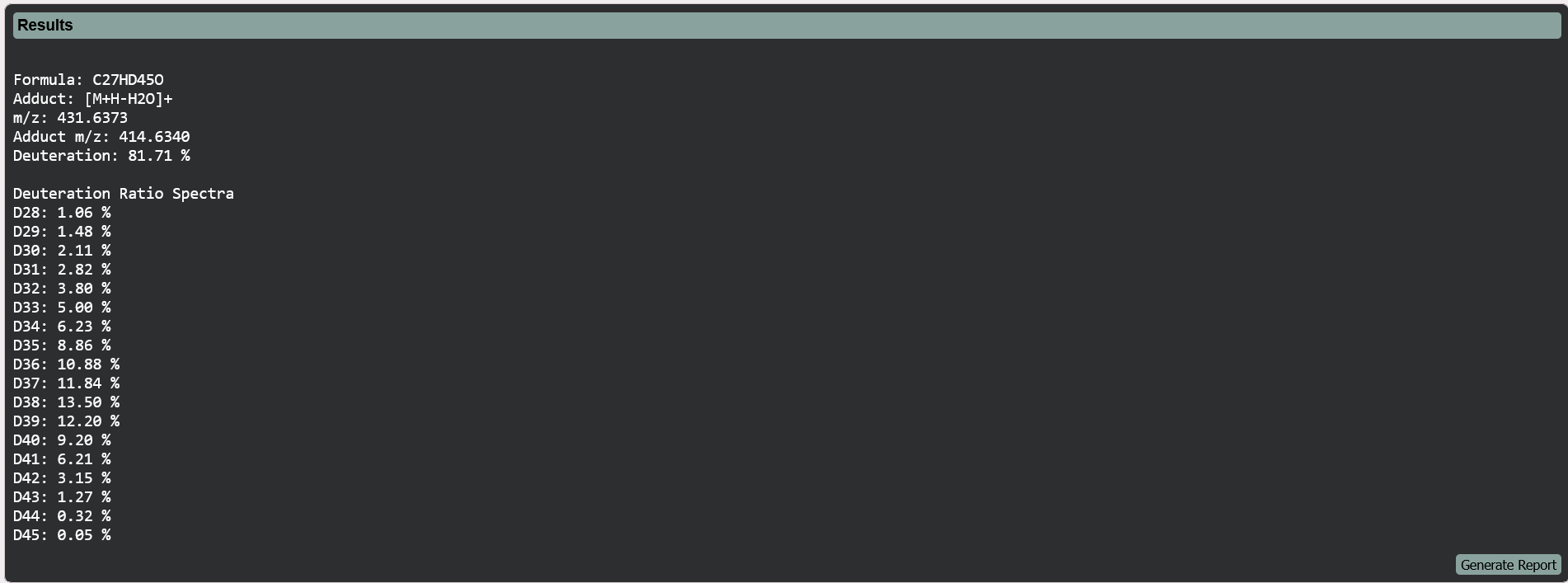


Figure S8: Location of the “Generate Report” button (bottom right) which can be used to generate a report of the DGet! analysis


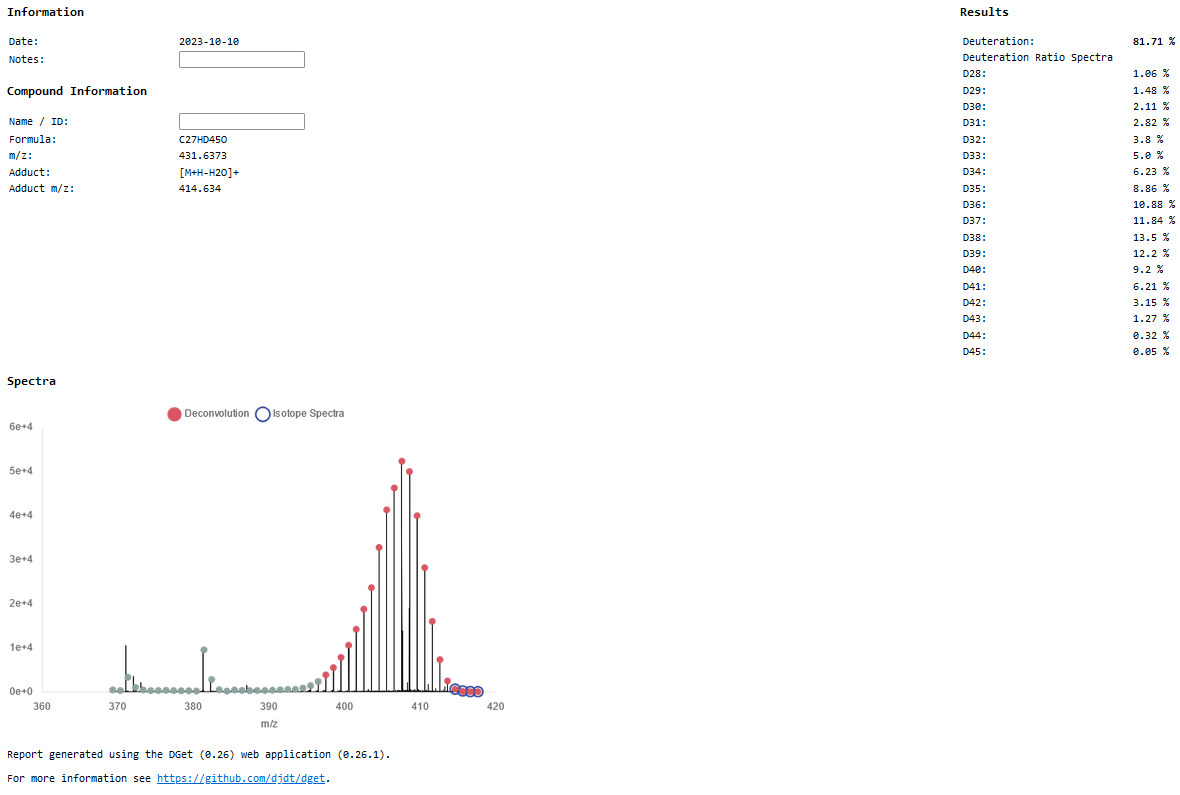

Figure S9: The report generated after using “Generate Report”

Lastly, there are a series of links at the bottom of the web app which direct the user to various related content:

- ‘GitHub’ will take the user to the DGet! GitHub repository.
- ‘Reference’ will take the user to ‘Read The Docs’ repository which contains information on installing and using DGet!, and full documentation of the Python code (<https://dget.readthedocs.io/en/latest/>).
- ‘Help’ contains information about DGet! (Figure S10).


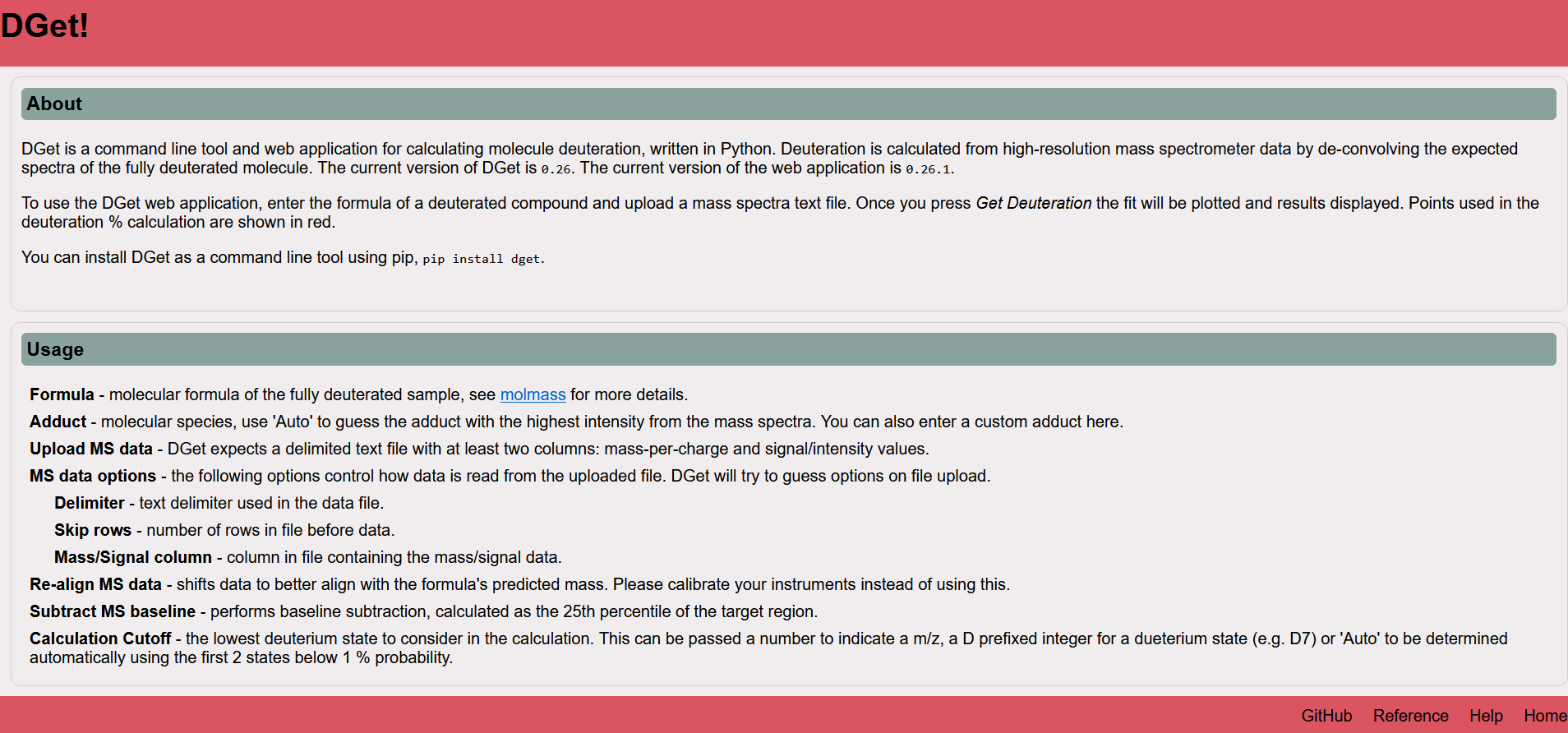


Figure S10: Contents of the ‘Help’ page.
